# New Insights into Materials for Pesticide and Other Agricultural Pollutant Remediation

**DOI:** 10.3390/ma17143478

**Published:** 2024-07-13

**Authors:** Afonso Henrique da Silva Júnior, Júlia de Oliveira Martins Müller, Carlos Rafael Silva de Oliveira, Agenor de Noni Junior, Robert Kimutai Tewo, Washington Mhike, Adriano da Silva, António Benjamim Mapossa, Uttandaraman Sundararaj

**Affiliations:** 1 Department of Chemical Engineering and Food Engineering, Federal University of Santa Catarina, Florianópolis 88040-900, SC, Brazil; afonso.silva@posgrad.ufsc.br (A.H.d.S.J.); julia.omm@posgrad.ufsc.br (J.d.O.M.M.); carlos.oliveira@ufsc.br (C.R.S.d.O.); agenor.junior@ufsc.br (A.d.N.J.); adriano.silva@ufsc.br (A.d.S.); 2 Department of Textile Engineering, Federal University of Santa Catarina, Blumenau 89036-256, SC, Brazil; 3 Department of Chemical Engineering, Dedan Kimathi University of Technology, Kiganjo/Mathari, B5, Dedan Kimathi, Nyeri Private Bag 10143, Kenya; kimutaitewo@gmail.com; 4 Polymer Technology Division, Department of Chemical, Metallurgical and Materials Engineering, Tshwane University of Technology, Pretoria 0183, South Africa; mhikew@tut.ac.za; 5 Department of Chemical and Petroleum Engineering, University of Calgary, 2500 University Drive NW, Calgary, AB T2N 1N4, Canada

**Keywords:** emerging contaminants, heavy metals, agrochemicals, adsorption, catalytic processes

## Abstract

The increase in the world population and the intensification of agricultural practices have resulted in the release of several contaminants into the environment, especially pesticides and heavy metals. This article reviews recent advances in using adsorbent and catalytic materials for environmental decontamination. Different materials, including clays, carbonaceous, metallic, polymeric, and hybrid materials, are evaluated for their effectiveness in pollutant removal. Adsorption is an effective technique due to its low cost, operational simplicity, and possibility of adsorbent regeneration. Catalytic processes, especially those using metallic nanoparticles, offer high efficiency in degrading complex pesticides. Combining these technologies can enhance the efficiency of remediation processes, promoting a more sustainable and practical approach to mitigate the impacts of pesticides and other agricultural pollutants on the environment. Therefore, this review article aims to present several types of materials used as adsorbents and catalysts for decontaminating ecosystems affected by agricultural pollutants. It discusses recent works in literature and future perspectives on using these materials in environmental remediation. Additionally, it explores the possibilities of using green chemistry principles in producing sustainable materials and using agro-industrial waste as precursors of new materials to remove contaminants from the environment.

## 1. Introduction

In recent years, the intensification of agricultural practices on the planet has been causing numerous consequences for the environment, such as the release of contaminants from agrochemicals and fertilizers applied to crops [1]. This situation is not just a concern for the future but a pressing issue that demands immediate attention. The main cause of this intensification is attributed to the increase in the number of the world’s population, which is at approximately 8 billion. According to the Food and Agriculture Organization of the United Nations (FAO), the estimated global population in 2050 is 10 billion people [2]. However, based on the current number of people and the increase in the population growth rate each year, this estimate may be reached earlier than expected. Thus, the agricultural sector is and will be an even more important segment to sustain the possible significant number of the planet’s population in the coming decades [3].

In this scenario, the agricultural sector must support the enormous number of people globally, mainly by meeting the demand for food. However, to increase food production, it is necessary to intensify the application of agricultural inputs, such as fertilizers [4]. Furthermore, the emergence of new pathogens that affect plant development has been promoting the use of agrochemicals with formulations that are increasingly harmful to humans and the environment [5]. Although there is a trend towards using natural products, the intense use of synthetic agricultural pesticides remains. Therefore, the development of new products and processes that promote a reduction in the use of agricultural pesticides and that contribute to the environmental remediation of contaminants present in nature from agriculture can provide interesting alternatives to be applied [6].

The contamination of water resources and soil is a topic that deserves a lot of attention, since the maintenance of these ecosystems is fundamental to life on Earth. However, there are numerous processes that can be applied to remove contaminants from water and soil, offering a ray of hope in the face of environmental challenges. For example, adsorptive and catalytic processes are highly efficient alternatives used to remove emerging contaminants from the environment [7]. The advantages of adsorptive processes are numerous, such as low cost, ease of the production of adsorbents, simple operation, the possibility of using waste to prepare coal, low energy consumption, and application for the environmental remediation of numerous organic and inorganic compounds [8]. In addition, it is possible to reuse the adsorbents, which is a very positive point, and the adsorptive processes can be directed towards a treatment that presents economic and ecological viability. Catalytic processes, on the other hand, involve the acceleration of chemical reactions that transform contaminants into less harmful or easier-to-remove substances [9]. Heterogeneous catalysts, such as the nanoparticles of noble metals and metal oxides, are often used due to their high activity and selectivity [10,11]. These processes are highly effective in the degradation of complex and persistent pesticides, often resulting in the complete mineralization of the contaminants. The combination of catalytic processes with other treatment techniques, such as adsorption, can significantly increase the efficiency of remediation, making it a promising approach for the decontamination of water and soil [12].

**Figure 1:**
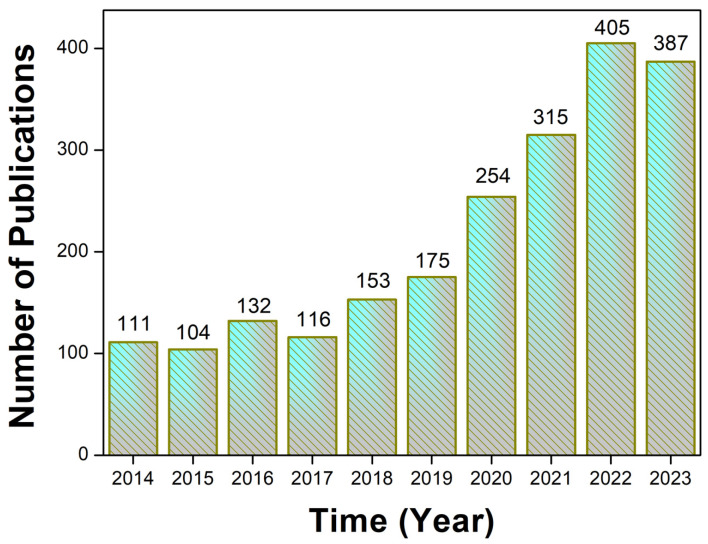
Number of publications from 2014 to 2023 with the keyword “Pesticide Remediation”.

The use of technologies and new materials for environmental remediation is a topic that has been widely explored by scientists and has been improved over the years on a laboratory scale [13,14]. More and more investments are being made to implement this in real water and effluent treatment plants. Thus, applying efficient techniques such as adsorptive and catalytic processes to remove contaminants from agricultural practices is an alternative that can significantly contribute to reducing the negative impacts caused by agriculture [1,15,16]. In the last ten years, numerous studies have been reported on treating ecosystems contaminated with pesticides. Figure 1 illustrates the number of publications from 2014 to 2023 with the keyword “Pesticide Remediation” in the Scopus database. In the last five years, the number of reported studies has been more than half of the total number of publications. Therefore, this review article aims to present several types of materials used as adsorbents and catalysts for ecosystems contaminated with pollutants from crops, as well as discuss some recent works in the literature and the limitations and future perspectives on using these materials in environmental remediation. In addition, the purpose of this review is to present the possibilities of using green chemistry principles in the production of sustainable materials and using agro-industrial waste as precursors of new materials to remove contaminants from nature. Comprehensive searches were conducted in the Scopus database to develop this review article on materials used as adsorbents and catalysts in ecosystems contaminated by agricultural pollutants. The keywords employed included adsorbents, catalysts, agricultural pollutants, environmental remediation, green chemistry, sustainable materials, and agro-industrial waste. The selection of references was based on a meticulous review of titles and abstracts, with the objective of including publications that aligned with the scope of the study within the past five years.

## 2. Pesticides and Their Negative Effects

Pesticides are chemicals widely used in agriculture to control pests, diseases, and weeds that affect crops [17]. Although pesticides play a crucial role in agricultural productivity and food security, they pose significant risks to human health and the environment. Pesticide exposure can occur in various ways, including ingesting contaminated food, inhaling airborne particles, and direct skin contact. Adverse effects on human health vary depending on the type of pesticide, dose, and duration of exposure. Among the main negative impacts are endocrine problems, as many pesticides can interfere with the human hormonal system, causing imbalances that can result in reproductive problems, abnormal sexual development, and increased risk of specific cancers [18]. In addition, organophosphate and carbamate pesticides are notorious for affecting the nervous system, causing symptoms ranging from headaches and dizziness to seizures and, in extreme cases, death [19]. Epidemiological studies have also linked exposure to pesticides to an increased risk of developing several types of cancer, including leukemia and cancers of the breast, prostate, lung, and skin. Acute exposure to high levels of pesticides can result in immediate poisoning, with symptoms such as nausea, vomiting, abdominal pain, breathing difficulties, and, in severe cases, death. On the other hand, chronic exposure to low levels can lead to long-term health problems, such as respiratory disease, liver and kidney damage, and immune disorders [20].

Pesticides also cause considerable damage to the environment, affecting biodiversity, water quality, and the health of ecosystems. Pesticides can contaminate water resources by leaching into groundwater or by chemical residues carried by rain to rivers, lakes, and oceans [21]. This results in the contamination of drinking water sources and a threat to aquatic life, as organisms such as fish, amphibians, and invertebrates are especially vulnerable and may suffer mass die-offs and a reduction in biodiversity. Furthermore, pesticide application can affect target species and non-target organisms, such as pollinators, that are essential for the reproduction of many plants, including bees and butterflies. The bioaccumulation and biomagnification of pesticides in the food chain can adversely affect top predators, such as mammals [22].

**Figure 2:**
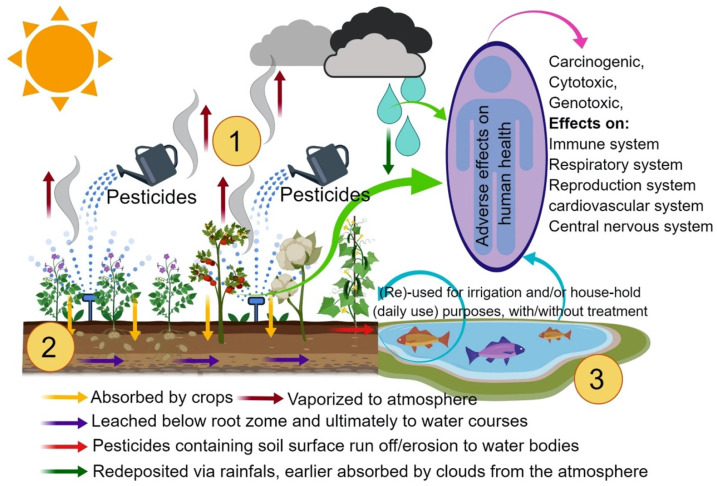
Negative impacts of soil, air, and water contamination on the environment and humans by pesticides. Reproduced with permission from [25].

Soil degradation is another significant environmental effect of pesticides, as these products can alter the soil microbiota, reducing its fertility and ability to support plant life [23]. Soil degradation compromises agricultural productivity in the long term and results in the loss of organic carbon, vital for soil structure and health. In addition, continued and intensive use of pesticides can lead to resistance in pest populations, requiring ever-higher doses of pesticides or the development of new chemical compounds, perpetuating a vicious cycle of increasing agrochemical use [24]. Figure 2 illustrates some of the negative impacts of soil, air, and water contamination on the environment and humans by pesticides.

Given these negative impacts, it is essential to develop integrated pest management strategies, adopt sustainable agricultural practices, and continue researching less harmful alternatives, such as biopesticides and biological control methods [6,26]. These approaches are essential to minimize the adverse effects of pesticides on human health and the environment, promoting safer and more sustainable agriculture. In addition, there are advanced alternatives for treating water and soil contaminated with pesticides, such as adsorption and heterogeneous catalysis. Both techniques are highly efficient and can be applied using environmentally friendly materials. Some classes of materials applied in treating ecosystems contaminated with pesticides and other agricultural pollutants are presented and discussed below.

## 3. Materials Used in Ecosystems Contaminated with Pesticides and Other Agricultural Pollutants

**Figure 3:**
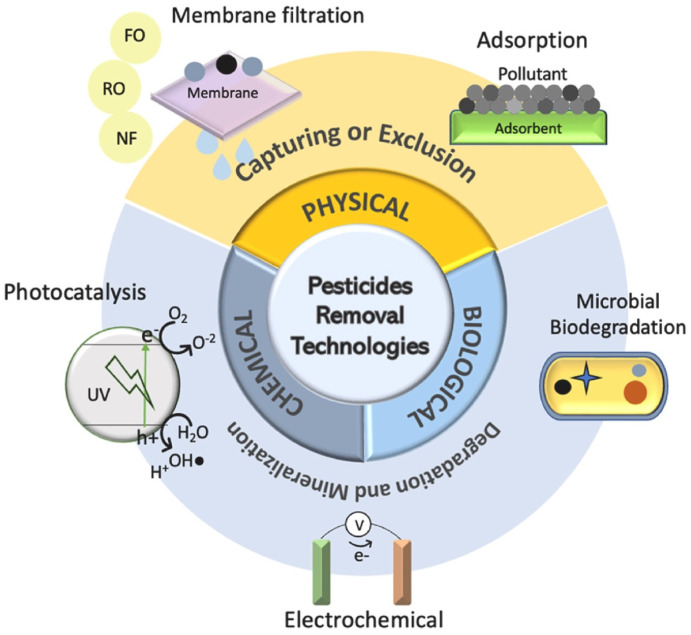
Approaches used for removing pesticides from contaminated ecosystems. Reproduced with permission from [29].

Several inputs necessary for plant development, such as pesticides and metal-based fertilizers, are used in agriculture [1]. Unfortunately, a large part of the load applied to fields is lost to the environment due to the excesses practiced in agricultural management. Commodities, non-industrialized global essential products, mainly grains, are some of the products to which large amounts of pesticides are applied in agriculture [4]. In Brazil, soybeans, corn, and sugarcane consume more than half of the total of agricultural pesticides sold in the country. Pesticides can be applied directly to plants or soil. However, half of the product ends up in water resources and soil due to losses [27]. The presence of metals from agricultural fertilization and pesticides in water is also alarming. Thus, removing these pollutants has been crucial for maintaining living organisms. Therefore, adsorptive and catalytic processes are options for removing these contaminants emerging from nature. Decontaminating ecosystems polluted with pesticides and other agricultural pollutants is a significant environmental challenge. Developing effective materials for this purpose has been a primary focus of scientific research [28]. Several materials and processes have been applied to remove pesticides and other agricultural pollutants from water and soil. Figure 3 illustrates some approaches used to remove pesticides from contaminated ecosystems. Table 1 shows the feasibility, cost-effectiveness, and affordability of different approaches to removing agricultural pollutants from contaminated ecosystems.

**Figure 4:**
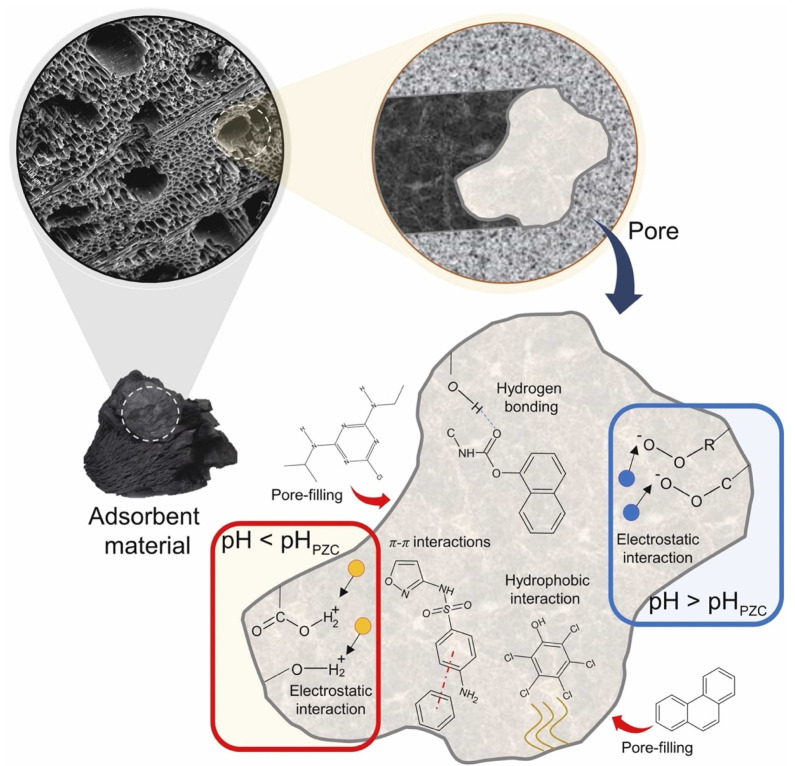
Reaction mechanisms in the removal of pollutants by biochar. Reproduced with permission from [2].

Adsorbents are materials that remove contaminants through physicochemical mechanisms, where pesticide molecules adhere to the material’s surface. The most-used adsorbents are activated carbon, biochar, zeolites, and carbon nanomaterials, such as carbon nanotubes and graphene [34,35]. Activated carbon is widely used due to its high surface area and porosity, efficiently removing many pollutants. It is often used in water treatment systems because it adsorbs organic and inorganic compounds. Biochar, produced by biomass pyrolysis, is a sustainable and low-cost alternative [36]. In addition to being used as an adsorbent, biochar can improve soil quality by increasing its water and nutrient retention capacity. Figure 4 illustrates some reaction mechanisms in the removal of pollutants by biochar. The mechanisms that control pollutant adsorption may be classified into different types, including electrostatic, hydrophobic, pore-filling, hydrogen bonding, and π–π interactions.

Zeolites, microporous minerals with a crystalline structure, allow the selective adsorption of pesticides [37]. Both natural and synthetic zeolites have shown efficacy in removing several contaminants, including organochlorine and organophosphate pesticides. Carbon nanomaterials, such as carbon nanotubes and graphene oxide, are advanced adsorbents with a high surface area and affinity for organic compounds, effectively removing pesticides due to their unique physical and chemical properties [38]. The adsorbents can be produced through different methodologies and precursors; for example, they can be hydrothermally produced with a metal–organic structure [39]. Therefore, strategies may be applied to synthesize adsorbents and, thus, adapt them to the purpose of the application. Nowadays, several published works use agro-industrial waste or clay-based materials to produce adsorbents to remove pollutants from water and soil [40]. In this sense, the precursors for manufacturing adsorbents are diverse and can be chosen based on the application. Thus, the choice of the precursor and the methodology for preparing the adsorbents are fundamental steps to achieve success in the application since the composition of each material and the morphology of each structure produced can contribute to satisfactory results depending on the contaminant.

On the other hand, catalytic processes involve the use of materials that accelerate the degradation of organic contaminants into less toxic substances. Heterogeneous catalysts, particularly the nanoparticles of noble metals and metal oxides, have emerged as key players in this field [9]. Nanoparticles of metals such as platinum, palladium, and gold are utilized in advanced oxidation reactions, demonstrating high efficiency in the degradation of complex and persistent pesticides, often leading to their complete mineralization. Metal oxides, including titanium, zinc, and iron, are extensively researched for their photocatalytic properties [41]. When exposed to UV light, these materials can generate free radicals that degrade pesticides into less harmful products. Titanium dioxide is widely recognized for its chemical stability and high photocatalytic activity, making it a popular choice in this context.

### 3.1. Materials Based on Agro-Industrial Waste

**Figure 5:**
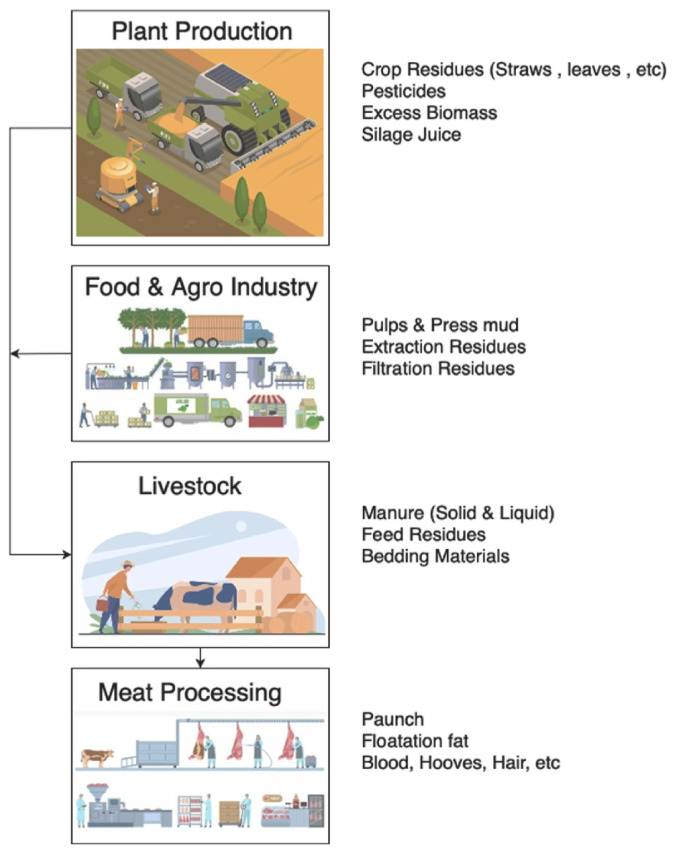
Types of agro-industrial waste and their origins. Reproduced with permission from [44].

Using waste to manufacture adsorbents or catalytic supports is a promising strategy due to its low production cost, easy handling, and availability, and because it solves a significant problem: improper disposal [42]. Agricultural waste is generated in large quantities and can be toxic to the environment when exposed to the weather for a long time. Generally, agricultural waste has a high carbon content that, after appropriate modifications, can be converted into high-performance activated carbon. In addition, numerous studies in the literature show that some low-cost materials produced from agricultural waste have greater efficiency when compared to commercial carbon [43]. Figure 5 illustrates some types of agro-industrial waste and their origins used in producing materials to remediate contaminated ecosystems. The main residues from agricultural practices for synthesizing adsorbents are rice husks, sugarcane bagasse, corn cobs, and peanut shells.

The production of adsorbents from agro-industrial waste is a very viable alternative. However, modifications in the structure and composition, such as functionalization, are necessary for optimization during the application of the material. The production of adsorbents from sugarcane and cassava with physicochemical modifications was tested by removing a cationic dye [40]. The raw materials were treated with sodium hydroxide. In addition, the ashes were produced from carbonization and calcination. The synthesis of a new activated carbon using the pyrolysis of a mixture of coffee grounds, eucalyptus sawdust, calcium hydroxide, and soybean oil at 800 °C was also reported as a good adsorbent material for removing endocrine disruptors [45]. The synthesis of an adsorbent from unmodified lemon peel was reported as an excellent material for the adsorption of copper ions in aqueous solutions [42]. Boontongto and coworkers developed a novel eco-friendly magnetic material derived from biomass waste [46]. They synthesized this material using aqueous ethanol as a green porogen, minimal toxic compounds as template molecules, and biocompatible binary monomers (tyrosine and tryptophan). They evaluated the binding characteristics and selectivity towards pesticides, revealing high adsorption capacities (from 150.11 to 509.09 mg·g^−1^). They utilized the material to extract organophosphorus and carbamate pesticides before HPLC analysis. Under optimal conditions, they achieved low detection limits (0.05–1.49 μg·L^−1^) and quantitation limits (0.18–5.00 μg·L^−1^). This approach enabled the efficient analysis of vegetable and fruit samples, with recoveries between 80% and 110%.

Mustafa and coworkers prepared graphene, Na-alginate, and Fe_3_O_4_ composites with algal biomass to remove 2,4-dichlorophenoxyacetic acid (2,4-D) in batch and column modes [47]. They pre-treated de-oiled algal biomass (chlorella) and used it to create biocomposites. The optimal batch adsorption parameters included a pH of 2, 0.05 g biosorbent dosage, 303 K, 90 min contact time, and 50 ppm pesticide concentration. The H_2_SO_4_ pre-treated biomass, graphene/algal, Fe_3_O_4_/algal, and Na-alginate/algal biocomposites exhibited biosorption capacities of 19.73, 20.43, 21.34, and 19.2 mg·g^−1^, respectively, for 2,4-D removal. The adsorption data followed the Freundlich isotherm and pseudo-second-order kinetics models. The researchers optimized the flow rate, bed height, and initial pesticide concentration in the column mode, achieving 8.55 mg·g^−1^ 2,4-D removal at a 2 cm bed height and 50 ppm initial pesticide concentration. The desorption studies confirmed the recyclability of biocomposites. The results indicated that graphene, Na-alginate, and Fe_3_O_4_ composites with algal biomass are highly effective for 2,4-D adsorption and suitable for remediating 2,4-D from effluents.

The researchers prepared KOH-activated crayfish shell biochar (KBC) and demonstrated its effectiveness in removing acetamiprid (ACE) and triadimefon (TDM) from aqueous solutions [48]. Batch experiments revealed that ACE adsorption primarily occurred through chemical reactions and was governed by multiple mechanisms. The adsorption of ACE was found to be thermodynamically spontaneous and exothermic, with KBC achieving a maximum adsorption capacity of 40.41 mg·g^−1^. KBC showed superior adsorption capacity for both ACE and TDM at lower concentration levels and demonstrated good reusability. ACE and TDM adsorption mechanisms by KBC involved van der Waals forces, pore filling, hydrogen bonding, and π–π electron donor–acceptor interactions.

Given this context, the use of agro-industrial waste to produce adsorbents is an eco-friendly path to follow, especially in countries with enormous agricultural potential. Furthermore, using adsorbents from waste reduces the environmental impacts caused by the disposal of this load and the possibility of developing new materials for environmental remediation.

### 3.2. Clay-Based Materials

Clay-based adsorbents are a potential material class for removing contaminants in different systems [49]. The inherent advantages of these adsorbents are their high adsorption capacity and ion exchange. In addition, clay-based adsorbents are abundant on the planet. Natural clay minerals such as bentonite and kaolinite can be categorized according to their structure [50]. Generally, clay minerals’ high adsorption capacity originates from their surface structure’s negative charges. Modified and unmodified kaolinite clays were tested for the removal of lead [51]. Aluminum sulfate and activated carbon were used in the synthesis of modified kaolinite. Additionally, the production of hybrid nanocomposites with clay is recurrent in the literature. Fabricating an inorganic–organic hybrid by selectively modifying the negative external surface of halloysite clay with two different organosilanes was applied as novel and economical adsorbents for extracting inorganic pollutants from an aqueous solution [52]. Applying natural clays found in nature is a strategy widely used by researchers. Samples of natural clay (montmorillonite and calcareous clays) collected in Tunisia were applied to remove various inorganic contaminants [53].

Researchers prepared composites of layered double hydroxides (LDH) modified with montmorillonite, mica, illite, and chlorite (LDH@Mt, LDH@Mi, LDH@Ill, and LDH@Chl) for the removal of atrazine (ATZ) and 4-chloro-2-methylphenoxyacetic acid (MCP) [54]. Kinetic studies showed that the adsorption processes for all four composites followed pseudo-second-order kinetics and intraparticle diffusion models. The generalized Langmuir model provided a better fit for the adsorption data than the Langmuir model, suggesting the presence of heterogeneous adsorption sites for ATZ and MCP. LDH@Mt exhibited superior adsorption performance with 8.19 mg·g^−1^ capacities for ATZ and 90.9 mg·g^−1^ for MCP. The site energy distribution theory qualitatively analyzed the number of adsorption sites, indicating that LDH@Mt had more accessible sites than other adsorbents. Thermodynamic analysis revealed that the removal processes were endothermic and spontaneous.

Boukhemkhem and coworkers studied the feasibility of using low cost and natural bentonite and kaolinite clays, compared with commercial bentonite, as raw materials to prepare catalysts for the removal of two emerging pollutants, the neonicotinoid pesticides nitenpyram and acetamiprid, by the heterogeneous Fenton process [55]. The raw materials were subjected to a simple ion exchange method to improve their textural properties using a solution containing ferric cations followed by calcination at 500 °C. The results showed high activity for all of the catalysts in removing these pesticides. Additionally, stability tests were carried out, confirming the high stability of these materials. Therefore, clay-based adsorbents are potential materials for environmental decontamination. However, few studies have explored their application in depth and on a large scale in removing contaminants from agriculture in different ecosystems.

### 3.3. Carbonaceous Materials

**Figure 6:**
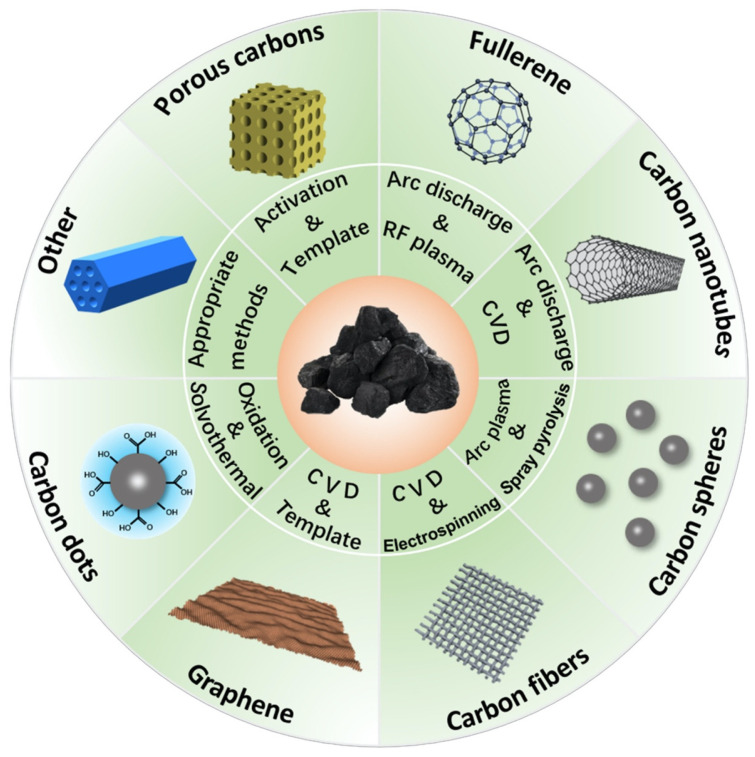
Types of carbonaceous materials and synthesis methodologies. Reproduced with permission from [61].

Carbonaceous materials are all those with a carbon-rich structure. Carbon nanoparticles have been analyzed for the treatment of contaminated water since they can form a complex hybrid material or be used in their pure form. Graphene, graphene oxide, activated carbon, single-walled and multi-walled carbon nanotubes, carbon quantum dots, and fullerenes stand out among the types of carbonaceous materials [56,57]. Figure 6 illustrates some types of carbonaceous materials and synthesis methodologies.

Two hybrid materials, resulting from the combination of carbon quantum dots, the metal–organic framework, and iron (Fe-CDs/MOF-808 and Fe-CDs@MOF-808), were developed aiming at the degradation of organophosphate pesticides [58]. Fe-CDs/MOF-808 was applied for the catalytic hydrolysis of paraoxon, promoting its total degradation after 5 min of reaction with a catalyst concentration of 0.5 mg∙mL^−1^. In the case of Fe-CDs@MOF-808, the material was analyzed for its ability to degrade parathion through photocatalytic oxidation. As a result, it was obtained that the degradation rate reached 98.1% after 10 min of reaction and that the contaminant was completely degraded after 30 min. According to a study by Boruah, Darabdhara, and Das (2021), graphene, functionalized with polydopamine and magnetite, may detect and degrade the pesticide simazine through photocatalysis [59]. The photocatalytic activity of the material was investigated under different light sources, with solar irradiation showing the maximum degradation efficiency (97%) after 50 min of reaction. Given this, four other triazine pesticides were analyzed: simetone, atrazine, ametryn, and prometryn, obtaining 89%, 87%, 95%, and 92% photodegradation efficiency. These results show that the developed material is suitable for the photocatalytic degradation of different pesticides in an aqueous medium; in addition, its efficiency remains high (76%) even after 12 cycles of use, demonstrating its potential for reuse. The functionalization of carbonaceous materials was also used to develop nanocatalysts from carbon nanotubes. The nanocatalysts obtained were analyzed for their catalytic activity in the degradation reactions of the pesticides diethyl 2,4-dinitrophenyl phosphate (DEDNPP) and diethyl 4-nitrophenyl phosphate [60]. The experiments demonstrated that, among the six catalysts produced, only two presented significant catalytic activities for the degradation of the simulated aqueous sample (DEDNPP), and these were used to treat a sample containing the pesticide paraoxon. Finally, the authors found that using the catalysts reduced the degradation time of paraoxon from 900 decades spontaneously to 9 and 10 h.

Carbon nanotubes were also used to produce two new adsorbent materials functionalized with surface groups, a carboxyl (-COOH) and a hydroxyl (-OH), and doped with magnetite [62]. The adsorption capacity was tested for removing atrazine and 2,4-dichlorophenoxyacetic acid (2,4-D) pesticides in simulated aqueous matrices and under real wastewater conditions. The hydroxyl-functionalized carbon nanotube (MWCNT-OH-Mag) showed the highest adsorption capacity for both pesticides, 51.4 mg∙g^−1^ and 47.7 mg∙g^−1^, for 2,4-D and atrazine, respectively. In addition, this adsorbent also had a higher regeneration efficiency after eight cycles of use. Both successfully degraded the pesticides when applied to real aqueous samples, with MWCNT-OH-Mag again showing the best results. Through this study, it was verified that both MWCNT-OH-Mag and MWCNT-COOH-Mag are promising adsorbents for the removal of recalcitrant pesticides from the aqueous medium. Carbon nanotube-based adsorbents are materials with the potential to remove contaminants from nature. Carbon nanotubes are considered remarkable due to their numerous characteristics, such as high porosity, accessible micropores, low weight, tunable surface properties, high corrosion resistance, well-defined structures, easy chemical activation, hollow internal structure, layered structure, and hydrophobicity [63]. Oxidized carbon nanotubes that showed efficient adsorption of cadmium have been reported [64]. This material was prepared through the thermal decomposition of methane in a hydrogen flow at a temperature of 750 °C using nickel nanoparticles as catalysts.

Mishra and coworkers synthesized a bifunctional In_2_S_3_/MgTiO_3_/TiO_2_@N-CNT (IMTNC) nanocomposite for sensing and removing clothianidin from aquatic environments via synergistic adsorption and photodegradation [65]. Structural, optical, morphological, and chemical characterizations confirmed the synthesis of IMTNC. The nanocomposite achieved a scavenging efficiency of 98.06% for ten ppm pollutant in 18 min. Incorporating In_2_S_3_ and N-doped carbon nanotubes (N-CNTs) improved the conductivity and electrochemical sensing, enhancing the charge transfer and photocatalytic activity.

Activated carbon may be synthesized from different raw materials, such as sewage sludge. Sanz-Santos and coworkers (2022) used five different types of sewage sludge to obtain the activated carbons [66]. They were later analyzed for their ability to remove the pesticides acetamiprid, thiamethoxam, and imidacloprid by adsorption. Among all of the activated carbons synthesized, the one obtained from industrial sludge showed the highest adsorption capacity with 104.2 mg∙g^−1^, 137 mg∙g^−1^, and 119.9 mg∙g^−1^ for acetamiprid, thiamethoxam, and imidacloprid, respectively. The recovery values of the materials ranged from 76% for industrial activated carbon to 95% for activated carbon from sludge from a paper mill. Industrial activated carbon, considered the best adsorbent, was applied to treat hospital effluent spiked with the three contaminants. As a result, the adsorbent showed high removal efficiencies in the macroscopic parameters of the effluent and a significant decrease in the removal of pesticides due to the medium’s complexity.

Hydrochar, obtained by the hydrothermal carbonization of biomass, is also an adsorbent material capable of removing pharmaceutical compounds, personal hygiene products, chemicals, pesticides, and microplastics [67]. Due to its potential, Duran, Bayarri, and Sans (2024) have synthesized a hydrochar from vineyard pruning wastes intending to adsorb five different types of pesticides: clothianidin (CTD), acetamiprid (ACE), 2,4-D, metalaxyl (MET), and atrazine (ATZ) [67]. The optimized conditions, 100 mg of adsorbent, pH 7, room temperature, and pesticide concentration of 20 µmol·L^−1^, provided a removal percentage of 85% for CTD, 94% for ACE, 86% for MET, 95% for ATZ, and only 4% for 2,4-D. These results clearly show the possibility of using this material to control environmental pollution caused by pesticides and valorize biomass residues.

Nassar and coworkers developed a hydrochar with iron nanoparticles from banana peels [68]. This material was used to remove three organophosphate insecticides: ethoprophos (ETH), terbufos (TER), and diazinon (DIA). Based on the experiments, the ideal conditions for the adsorption of these pesticides were 70 mg of adsorbent, pH 4, temperature of 25 °C, insecticide concentration of 1.5 ppm, and contact time of 3 h. By analyzing the adsorption isotherms, the experimental data were best adjusted by the Langmuir model, resulting in a maximum adsorption capacity of 3.19, 3.07, and 3.19 mg·g^−1^ for ETH, TER, and DIA, respectively. Hydrochar provided a removal efficiency above 95% for all of the compounds analyzed. In addition, it has an excellent recovery capacity, obtaining removal percentages more significant than 80% even after seven cycles of use.

Furthermore, hydrochar may also be obtained from the bark of *Prunus serrulata*, as demonstrated by Netto and coworkers [69]. The authors used the hydrochar produced to remove the herbicide atrazine, obtaining a maximum adsorption capacity of 63.35 mg·g^−1^ in 240 min, 328 K, pH 3, atrazine concentration of 50 ppm, and an adsorbent dosage of 0.8 g·L^−1^. This material was also analyzed for its removal capacity of this compound in three contaminated river samples, which allowed removal percentages above 70% for all three cases.

In addition, the fabrication of hybrid adsorbents is a solution widely applied in the literature due to improved properties and characteristics, such as the increase in surface area. A mesoporous organosilica hybrid integrated with disulfide and amine groups was synthesized by a sol–gel co-condensation method using Pluronic P123 [70]. The hybrid was applied in the simultaneous adsorption of Hg^2+^ and Cu^2+^ ions from aqueous solution. Additionally, carbon-based magnetic materials can be applied in the adsorption of pollutants [71]. Therefore, there is a wide variety of types of materials that can be used in the synthesis of adsorbents. Thus, designing methodological strategies and applying experimental designs can be an interesting path to follow for applying adsorbent materials in environmental remediation.

### 3.4. (Bio)polymer-Based Materials

Bakka and coworkers (2020) introduced a novel biosorbent, developed from the chemical treatment of the external shells of marine gastropods [72]. Their experiments on the adsorption of the insecticide bifenthrin in a fixed bed column confirmed the effectiveness of this unique biomaterial. The characterization and experiments revealed that the biosorbent had a large specific surface area and that the pesticide adsorption was enhanced by increasing the flow, bed height, feed concentration, and decreasing particle size. Under the optimal conditions, the biosorbent demonstrated the ability to remove 40.53 mg of the pesticide and showed a high regeneration potential, maintaining good efficiency even after five cycles of use.

Mostafa and coworkers (2021) developed a hybrid adsorbent, integrating chitosan and zeolite-A, to remove organophosphate pesticides [73]. This innovative material exhibited an impressive adsorption capacity of 650.7 mg∙g^−1^, 506.5 mg∙g^−1^, and 560.8 mg∙g^−1^ for acephate, omthosate, and methyl parathion, respectively. Furthermore, the hybrid adsorbent demonstrated a good regenerative capacity, maintaining removal values above 90% after five cycles of use for the three pesticides analyzed.

Motaghi and coworkers (2022) also investigated the application of chitosan as an adsorbent [74]. In this case, the authors proposed the development of a bio-nanocomposite, which was obtained by modifying the composite formed by magnetic chitosan and activated carbon (MCS/AC) with metal–organic frameworks (MOFs). The biomaterial was analyzed for its ability to remove cobalt (II) ions, azo dye, and the pesticide imidacloprid. The maximum adsorption capacity for the pesticide was 25.23 mg∙g^−1^ in a contact time of 15 min. Furthermore, applying this material promoted removals above 90% for real samples, and, after five cycles of use, the adsorption efficiency was 88.45%. Due to its distinct properties, chitosan has also been used with graphene quantum dots and iron oxide nanoparticles to obtain an adsorbent material capable of removing the pesticide chlorpyrifos. This research analyzed the influence of the amount of graphene quantum dots and iron oxide nanoparticles (GQD-Fe_3_O_4_) on the pollutant removal efficiency [75]. The results demonstrate that adding 10% of GQD-Fe_3_O_4_ to the chitosan solution and using optimized parameters promotes maximum adsorption efficiency (99.9%) after 50 min of contact. However, this time may be reduced to 10 min by applying ultrasound. The total amount of pesticide adsorbed for the experiments operated in continuous flow was 24.72 mg∙g^−1^. In the end, it was found that the material developed based on chitosan promotes good adsorption of the target compound, even in complex media. It can be reused for up to seven cycles without significantly decreasing adsorption efficiency.

In addition to the forms exposed, biomaterials can be obtained from biomass residues or byproducts. In this sense, Deyris and coworkers (2023) developed biosorbents from the powder of three dead roots of invasive species and coffee and green tea grounds [76]. The developed materials were tested to remove different types of emerging contaminants, including chlordecone, lindane, diuron, and ametryne, which belong to the group of pesticides. For chlordecone, lindane, and ametryne, the best removal was obtained by the adsorbent produced from the coffee grounds, resulting in removal percentages of 96%, 89%, and 59%, respectively. For the pesticide diuron, the maximum removal was 90% using the adsorbent produced by *Fallopia japonica* TT, an invasive plant species. When subjected to a reconstituted seawater sample, the biomaterials demonstrated a similar adsorption capacity for all of the pesticides analyzed.

Several studies have been carried out to obtain a biomaterial capable of acting as a photocatalyst in photocatalysis processes. Titanium dioxide is a widely used photocatalyst semiconductor due to its low cost, non-toxicity, and long-term stability. However, TiO_2_ is not a promising adsorbent, requiring a substrate, which can be a biomaterial capable of adsorbing pollutants and increasing the efficiency of photocatalysis [77]. Given this, Hasanin and coworkers (2021) developed a photocatalyst from the combination of carboxymethyl cellulose, tryptophan, and titanium dioxide [78]. The new nanocomposite was prepared for the degradation of the pesticide 2,4-dichlorophenol (2,4-DCP). The degradation rate obtained was 0.02061 min^−1^ for a dosage of 0.5 g∙L^−1^ of photocatalyst, which makes the developed material a good alternative since it presents the highest removal rate compared to the values reported in the literature.

### 3.5. Other Materials

An organic–inorganic hydrogel nanocomposite based on polyamidoxime grafted with gum arabic and magnetic CuFe_2_O_4_ nanoparticles was reported [79]. The adsorption efficiency of the nanocomposite for removing an organophosphate pesticide from aqueous solutions was tested. The effect of different experimental conditions, such as solution pH, adsorbent dosage, contact time, and initial concentration, on the adsorption efficiency was evaluated. The experimental adsorption data were well-fitted by the Langmuir isotherm model, and the maximum adsorption capacity of the prepared adsorbent was 769.23 mg∙g^−1^. The pseudo-second-order model fitted the adsorption kinetics data well. It was suggested that the pollutant adsorbed on the nanocomposite occurred through electrostatic interactions and hydrogen bonding. Furthermore, the adsorption–desorption experiments revealed that the adsorbent could be efficiently regenerated and reused after three sequential runs without a considerable decline in its adsorption performance.

Faisal and coworkers developed a novel ternary photocatalyst incorporating Pt nanoparticles, multi-walled carbon nanotubes (MWCNT), and ZnTiO_3_ nanostructures [80]. They synthesized ZnTiO_3_ using the sol–gel method and fabricated the Pt@MWCNT/ZnTiO_3_ photocatalyst via ultrasonication and photoreduction routes. X-ray diffraction analysis confirmed the formation of rhombohedral ZnTiO_3_, and transmission electron microscopy revealed a well-dispersed distribution of Pt in the MWCNT/ZnTiO_3_ nanocomposite. The photocatalyst demonstrated high efficiency, achieving 92.62% removal of the insecticide thiamethoxam in only 40 min under visible light, 2.12 times more efficient than pure ZnTiO_3_. The superior photodegradation capabilities of Pt@MWCNT/ZnTiO_3_ were attributed to the highly ordered mesoporous ZnTiO_3_, increased surface area, favorable bandgap, improved visible light absorption, and effective mitigation of charge carrier recombination.

Gadore and coworkers developed a novel SnS_2_/CO_3_^2−^@Ni-Co LDH (SnS_2_/NCL) nanocomposite to enhance the degradation of the insecticide thiamethoxam through advanced oxidation processes [81]. The optimized conditions achieved nearly complete (98.1%) degradation of the pollutant (10 ppm) with a rate constant of 0.0541 min^−1^ using 0.16 g·L^−1^ catalyst and 0.3 mM H_2_O_2_ under visible light for 70 min. The SnS_2_/NCL nanocomposite showed reduced degradation efficiency in the presence of most metal cations and inorganic anions except Fe^2+^, which increased the production of hydroxyl radicals. The material maintained 83.6% efficiency after five cycles and over 80% efficiency for other organic pollutants. Integrating Ni-Co LDH with SnS_2_ improved the photocatalytic activity by reducing the electron–hole recombination rates, demonstrating its potential for wastewater treatment.

**Figure 7:**
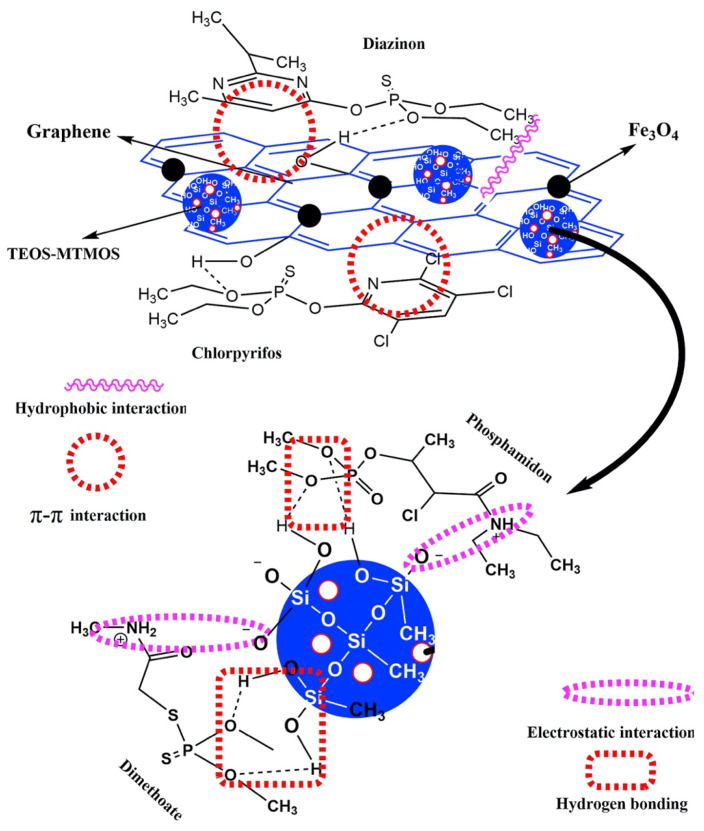
Proposed mechanism of pesticide adsorption by Fe_3_O_4_@G-TEOS–MTMOS. Reproduced with permission from [82].

A graphene-based sol–gel hybrid magnetic nanocomposite (Fe_3_O_4_@G-TEOS–MTMOS) was synthesized and successfully used in the magnetic solid-phase extraction for the analysis of organophosphorus pesticides in water samples [82]. Characterized by various techniques, including FTIR and XRD, it showed a high adsorption capacity and excellent extraction recoveries (83–105%) for pesticides in different water samples, with detection below the maximum levels allowed by the European Union. The polar selectivity of the adsorbent was tuned by dispersing inorganic–organic material on the graphene sheets. The graphene-based adsorbent was sensitive to nonpolar pesticides with benzene rings in its structure due to strong π−π stacking. TEOS–MTMOS conferred sensitivity to polar pesticides through hydrogen bonding and electrostatic interactions. Figure 7 illustrates a meticulously proposed mechanism of pesticide adsorption by Fe_3_O_4_@G-TEOS–MTMOS, delineating the intricate interplay of physicochemical forces at work.

The magnetic removal and recovery of heavy metals and pesticides from the soil by a novel biotite modified with a double hydroxide layer were recently reported [83]. The synergistic effect of the adsorbent precursors promoted the increase in the removal capacity of metal ions and agrochemicals. Through the kinetic experiments, it was observed that there was an excellent fit between the pseudo-second-order model and the intraparticle diffusion model. The Freundlich model described the isothermal experiments for copper and lead wells. For agrochemicals, the isothermal experiments were well-fitted by the Langmuir model. The removal mechanisms by the composite were precipitation for metal ions and surface adsorption for agrochemicals. In the column leaching experiment, the adsorbent material showed an effective interception of heavy metals and pesticides to prevent diffusion. In addition, the research suggested that the magnetic property of the adsorbent can avoid the potential risks of pollutants in the soil. An adsorbent produced from *Eucalyptus sheathiana* bark in its raw form and modified with sodium hydroxide was used to remove zinc from aqueous solutions [84]. The process was strongly pH-dependent, and the percentage of ion adsorption increased as the pH of the solution increased from 2.5 to 5.1. On the other hand, the percentage of zinc adsorption decreased with increasing adsorbent dosage, initial metal concentration, temperature, and ionic strength. The kinetic measurements showed that the process was multistep, fast, and diffusion controlled. Furthermore, it was found that the kinetics followed the pseudo-second-order equation. The equilibrium experiments showed that the Freundlich and Langmuir models applied to raw and modified eucalyptus bark.

Zhao and coworkers synthesized a tungsten boride iron phosphate (FePO_4_/WB) composite via a hydrothermal method to enhance Fe(III)/Fe(II) redox cycling for effective degradation of neonicotinoid insecticides (NEO) [85]. The FePO_4_/WB-PMS system generated radicals (HO^•^ and SO_4_^•−^) and nonradicals (^1^O_2_ and Fe(IV)), contributing 3.02%, 3.58%, 6.24%, and 87.16%, respectively, to the degradation of imidacloprid. Tungsten boride (WB) facilitated the reduction of FePO_4_, with Fe(II) primarily activating PMS via two-electron transfer to form Fe(IV) and a minor fraction producing SO_4_^•−^, HO^•^, and ^1^O_2_ via one-electron transfer. Four degradation pathways of NEO were identified using UPLC-Q-TOF-MS/MS. Seed germination tests indicated a significant reduction in NEO biotoxicity post-degradation. FePO_4_/WB showed high stability in recycling and continuous flow reactor experiments, offering a promising approach for water remediation via Fenton-type reactions.

Yari and coworkers examined the degradation and mineralization of imidacloprid in aqueous solutions using advanced oxidation processes: UVC, UVC/TiO_2_, and UVC/ZnO [86]. The experiments were conducted with a laboratory-scale batch photoreactor (100 mL) using a low-pressure mercury vapor lamp (254 nm). The optimal conditions for the UVC/TiO_2_ process (C_0_ = 100 ppm, pH = 7.5, t = 20 min, [TiO_2_] = 100 ppm) resulted in 88.15% insecticide degradation, following first-order kinetics. The degradation efficiency increased with the illumination time and was more favorable at alkaline pH. Comparing photocatalytic degradation with photolysis showed a significant synergy effect, with a 36.7% increase in pollutant removal efficiency in 20 min. GC/MS chromatograms confirmed that the UVC/TiO_2_ process effectively simplified the contaminant into more degradable compounds.

Beyond clay-based adsorbents, a vast array of other types of adsorbents exist, each with its unique properties and potential applications. Metal-based, carbonaceous, and hybrid materials are just a few examples [87]. The ligand-exchange fibrous adsorbent loaded with zirconium was used to adsorb arsenic from contaminated water [88]. The fibers, containing phosphonate and sulfonate groups, were synthesized through a complex process involving the graft polymerization of chloromethyl styrene on polypropylene fiber coated with polyethylene through electron irradiation. Another example is a porous conjugated material functionalized with a 4-nitro-1-naphthylamine ligand, which was prepared to efficiently monitor and remove nitrite from water samples [89]. Table 2 shows published studies that applied various materials to remove contaminants from agricultural practices.

The application of advanced materials in decontaminating ecosystems polluted by pesticides and other agricultural pollutants is a dynamic and vital research area for environmental sustainability. Adsorbents and catalysts are essential in providing effective solutions for removing pesticides, heavy metals, and other contaminants, each with specific advantages. The continued development of these technologies and the strategic combination of different approaches can lead to more efficient and sustainable solutions to mitigate the impacts of pesticides on the environment. This technological synergy not only improves the efficiency of decontamination processes but also contributes significantly to protecting natural resources and promoting a healthier environment.

## 4. Future Perspectives

The growing concern about the environmental impacts caused by the intensification of agricultural practices highlights the urgent need to develop practical materials and technologies to remove pesticides and other agricultural pollutants. This challenge has encouraged research in several areas, including creating new adsorbent and catalytic materials. Prospects in this field point to continuous innovation and sustainable solutions. A promising line of research involves the development of materials based on agro-industrial waste [99]. Exploring new wastes, such as banana peels or coffee grounds, to produce highly efficient biochar is an avenue to be explored in greater depth. In addition, chemical modification and functionalization of these materials can significantly improve their adsorption capacity. Chemical treatments, such as impregnation with metals or adding specific functional groups, can increase the efficiency of removing pollutants [57].

Hybrid materials and nanocomposites represent another area of great potential. The combination of properties of different materials, such as graphene with metal oxides, can create adsorbents with high adsorption capacity and advanced catalytic properties. Studying the synergies between these materials and their interactions with different types of pollutants can optimize the removal of multiple contaminants simultaneously. Table 3 presents a comparison of the material types discussed in this work, highlighting their respective advantages, disadvantages, and efficiencies in removing agricultural pollutants. Advanced catalytic processes, especially those using “green” catalysts, which are environmentally friendly and sustainable, are essential for the efficient degradation of complex pesticides [9]. The development of solar-activated photocatalysts integrated with adsorbents can maximize contaminant removal in real environments, offering a sustainable and effective solution.

The regeneration and reuse of materials are also crucial aspects. Developing efficient regeneration methods for adsorbents and catalysts will allow multiple use cycles without a significant performance loss. Performing life cycle analyses can help assess these materials’ sustainability and economic viability, promoting their large-scale application. Detailed investigation of the adsorption mechanisms of different pollutants on different materials is vital to optimize the selection of adsorbents based on the composition of the pollutants present in specific environments [2,36]. Modeling and simulation techniques can predict the behavior of new materials and their interactions with pollutants, accelerating the development of practical solutions.

The implementation of these technologies in water and wastewater treatment plants is a promising prospect. Integrating new materials and technologies into existing processes can increase the efficiency of contaminant removal. In addition, promoting sustainable agricultural practices that use fewer pesticides and fertilizers, such as crop rotation, organic farming, and integrated pest management, combined with remediation technologies, can minimize the environmental impact of agricultural activities. Finally, collaboration with policymakers to develop policies that encourage the use of sustainable technologies and continued research into environmental remediation materials is essential [101]. Nowadays, there are numerous specific regulations for the remediation of pesticides and other pollutants at contaminated sites. In the United States, the Environmental Protection Agency (EPA), the New Jersey Department of Environmental Protection (NJDEP), and the Tennessee Department of Environment and Conservation (TDEC) establish strict standards through various programs addressing the cleanup of sites contaminated with hazardous substances, including pesticides [2]. These regulations ensure that remediation is effective and sustainable, minimizing risks to public health and the environment. This comprehensive framework of regulations and policies promotes safe and efficient cleanup practices while fostering environmental sustainability. Advances in research and the development of new materials and technologies also are essential to address the environmental challenges arising from intensive agriculture. The synergy between different approaches and the implementation of innovative solutions will contribute significantly to the sustainability and protection of natural ecosystems. Therefore, some general trends may be observed:

- Continued advances in research should lead to new technologies and methods for pesticide remediation, including the development of innovative materials.
- There will be a persistent focus on developing sustainable and environmentally friendly remediation methods, emphasizing reducing energy consumption, waste generation, and carbon emissions.
- The understanding of microbial communities involved in pesticide degradation will deepen.
- Future research will likely focus on assessing the long-term risks associated with pesticide contamination.
- Advances in scientific knowledge may result in changes in regulations and policies on pesticide remediation and cleanup of contaminated sites.
- International collaboration and the exchange of best practices for pesticide remediation should increase, promoting a coordinated global approach.

## 5. Conclusions

In the coming decades, the world’s population is estimated to reach 10 billion people, making the agricultural sector even more crucial to society. However, agriculture’s growth must be conducted harmoniously, considering several aspects, such as the load of agricultural inputs applied to crops. Currently, experts are concerned about the imminent dangers that these compounds pose to living organisms and the preservation of the planet. In this context, applying adsorptive and catalytic processes to remove pollutants from the ecosystem is essential to reverse the negative impacts of human activities. This review sought to present some materials used in environmental decontamination, including agro-industrial waste, clays, carbonaceous, metallic, and hybrid materials. In addition, the main pollutants originating from agriculture, such as pesticides and metals, and the advantages of applying adsorptive and catalytic processes to remove these pollutants were discussed. Therefore, holding discussions based on experimental studies on the possible long-term consequences of these contaminants, reducing the massive application of agricultural pesticides, developing new products that are less harmful to ecosystems, deepening knowledge about the mechanisms involved in the adsorption process, and studying viable measures, both environmentally and financially, for the reuse of adsorbents are effective strategies for maintaining the balance and sustainability of the planet. Furthermore, raising awareness about developing environmentally friendly processes and applying new technologies are essential paths that future research should follow.

## Figures and Tables

**Table 1:** Feasibility, cost-effectiveness, and affordability of different approaches to removing agricultural pollutants from contaminated ecosystems.

Approach	Description	Feasibility	Cost–Benefit	Accessibility forStakeholders	Ref.
Physical	Methods such as adsorption, filtration, and membrane separation to remove pesticides from contaminated water	High for removing larger particles; limited for soluble compounds	Variable but may be expensive due to material and maintenance costs	Moderate; easy implementation, but cost may be a limiting factor	[30,31]
Chemical	Includes processes like advanced oxidation, chemical precipitation, and the use of chemical reagents to decompose pesticides	High efficiency in decomposing pesticides; requires strict control of reaction conditions	High efficiency but may be expensive due to reagent costs and the need for additional waste treatment	High for advanced oxidation processes; accessibility may be limited by the cost of chemical reagents	[31,32]
Biological	Uses living organisms (microorganisms and plants) to degrade pesticides into less toxic contaminants	Sustainable and environmentally friendly; depends on the presence of specific microorganisms	Generally cheaper and more sustainable in the long term; implementation can be complex	High; especially in agricultural areas where bioremediation may be integrated into farming practices	[33]

**Table 2:** Published works on the application of materials in the removal of pollutants from agricultural practices.

Material	Pollutant	Sample	Technology	Conditions	Efficiency	Ref.
Agro-industrial waste						
Biomass waste	Carbofuran, carbaryl, methiocarb, azinphos-ethyl, azinphos-methyl, parathion-ethyl, parathion-methyl, diazinon, fenitrothion, chlorpyrifos, and profenofos	Synthetic water and real samples	Adsorption	2 mg of sorbent material	CBFR: 150.11 mg·g^−1^; CBR: 421.99 mg·g^−1^; MTOC: 233.07 mg·g^−1^; AZM: 299.42 mg·g^−1^; PM: 459.36 mg·g^−1^; AZE: 482.17 mg·g^−1^; FNT: 395.30 mg·g^−1^; DZ: 351.52 mg·g^−1^; PE: 481.93 mg·g^−1^; PFF: 480.46 mg·g^−1^; CP: 509.09 mg·g^−1^. In 30 s	[46]
Algal biomass	2,4-dichlorophenoxyacetic acid	Synthetic water	Adsorption	0.005 g of biosorbent; pH: 2.0, 303 K	Fe_3_O_4_/Algal: 21.34 mg·g^−1^. In 90 min	[47]
KOH-activated crayfish shell biochar	Acetamiprid and triadimefon	Synthetic water	Adsorption	[Ads]: 1 g·L^−1^; 200 rpm; 298 K; pH: 6.0;	ACE: 38.88 mg·g^−1^; TDM: 26.77 mg·g^−1^. In 120 min	[48]
Soursop seeds and peanut shells	Cd^2+^ and Pb^2+^	Synthetic water	Adsorption	pH 5.0 [Ads]: 2 g·L^−1^ and 50 rpm	99%. In 3 min	[90]
Biomass of *Pseudomonas stutzeri* and *Delonix regia* seeds	Chlorpyrifos	Water and soil	Adsorption	1.5 g of bioadsorbent; 30 °C and pH 7.0	95.29% (Water); 93.10% (Soil). In 14 days	[91]
Rice bran, wheat bran, lentil hulls, and rice hulls	Cadmium	Synthetic water	Adsorption	[Ads]: 2 g·L^−1^, 30 °C and 120 rpm; pH 5.0	81%. In 60 min	[92]
Rice husk	Cd^2+^	Synthetic water	Adsorption	[Ads]: 0.5 g·L^−1^, 200 rpm and 288 K; pH 7.0	91.7%. In 120 min	[93]
Mango leaf	Methylene blue	Synthetic water	Adsorption	0.8 g_Adsorbent_, 350 osc·min^−1^ and 25 °C; pH: 7.0–10.0	99%. In 240 min	[94]
Clay-based materials						
Modified and unmodified kaolinite clay	Lead	Synthetic water	Adsorption	0.5 g_Adsorbent_, 150 rpm and 30 °C; pH 7.0	Modified kaolinite: 32.2 mg·g^−1^. In 60 min	[51]
Halloysite clay with two different organosilanes (P-HNTs and S-HNTs)	Hg(II)	Synthetic water	Adsorption	[Ads]: 3 g·L^−1^, 25 °C; pH 7.0	P-HNTs: 83.48 mg·g^−1^. In 60 min	[52]
Montmorillonite and calcareous clays (RS, TS, RY and TY)	Pb(II), Cd(II), Cu(II), and Zn(II)	Synthetic water	Adsorption	[Ads]: 1 g·L^−1^, 200 rpm; pH: 6.0	Pb(II): RS: 131.58 mg·g^−1^. Cd(II): TY: 16 mg·g^−1^. Cu(II): RS: 27.40 mg·g^−1^. Zn(II): RS: 22.73 mg·g^−1^. In 60 min	[53]
Double hydroxides (LDH) modified with montmorillonite, mica, illite, and chlorite (LDH@Mt, LDH@Mi, LDH@Ill, and LDH@Chl)	Atrazine (ATZ) and 4-chloro-2-methylphenoxyacetic acid (MCP)	Synthetic water	Adsorption	100 mg of adsorbent; 8000 rpm	LDH@Mt: ATZ: 8.19 mg·g^−1^; MCP: 90.9 mg·g^−1^. In 6 h	[54]
Natural bentonite and kaolinite clays	Nitenpyram (NTP) and Acetamiprid (ACP)	Synthetic water	Catalysis	0.2 g of catalyst; pH: 3.0; 55 °C. Pollutant: 5 mg·L^−1^.	Kaolin: 100%. In 180 min Bentonites: 100%. In 120 min	[55]
Carbonaceous materials						
Carbon quantum dots, metal–organic framework, and iron (Fe-CDs/MOF-808 and Fe-CDs@MOF-808)	Paraoxon and parathion	Synthetic water	Catalysis	Fe-CDs/MOF-808: 0.5 mg·mL^−1^; Paraoxon: 100 µM Fe-CDs@MOF-808: 0.5 mg·mL^−1^; Parathion: 100 µM	Paraoxon: Fe-CDs/MOF-808: 100%. In 5 min Parathion: Fe-CDs@MOF-808 100%. In 30 min	[58]
Graphene, functionalized with polydopamine and magnetite (FGD-10, FGD-15, and FGD-20)	Simazine, simetone, atrazine, ametryn, and prometryn	Synthetic water	Catalysis	Catalyst loading: 0.3 g·L^−1^, concentration of pesticides: 0.3 mM and pH 5.0; Sunlight irradiation	FDG-20: Simazine: 97%; Simetone: 89%; Atrazine: 87%; Ametryn: 95%; Prometryn: 92%. In 50 min	[59]
Carbon nanotubes (CNT, CNT01, CNT02, CNTIMZ1, CNTIMZ2, and RCNTIMZ)	Diethyl 2,4-dinitrophenyl phosphate (DEDNPP) and diethyl 4-nitrophenyl phosphate (Paraoxon)	Synthetic water	Catalysis	1 mg of the nanocatalyst, pH: 9.0	Reaction lifetime measured as 5 half-lives DEDNPP: RCNTIMZ: 55 s; CNTIMZ2: 47 s Paraoxon: RCNTIMZ: 9 h; CNTIMZ2: 10 h	[60]
Carbon nanotubes functionalized with surface groups (MWCNT–OH-Mag and MWCNT–COOH-Mag)	Atrazine and 2,4-dichlorophenoxyacetic acid (2,4-D)	Synthetic water and real water	Adsorption	[Ads]: 1 g·L^−1^, 200 rpm and 298 K; 2,4-D: pH: 2; Atrazine: pH: 6 (MWCNT–OH-Mag) and 8 (MWCNT–COOH-Mag); Pesticide: 50 ppm	2,4 D: MWCNT–OH-Mag: 51.46 mg·g^−1^; MWCNT–COOH-Mag: 49.97 mg·g^−1^. Atrazine: MWCNT–OH-Mag: 47.71 mg·g^−1^; MWCNT–COOH-Mag: 39.3 mg·g^−1^. In 30 min	[62]
In_2_S_3_/MgTiO_3_/TiO_2_@N-CNT (IMTNC)	Clothianidin	Synthetic water and real water	Adsorption and photodegradation	[Cat]: 0.313 g·L^−1^; pH: 5.34; 23 W LED/H_2_O_2_. Clothianidin: 10.85 ppm	98.06%. In 18 min	[65]
Activated carbon synthesized from sewage sludge (AC-Industrial, AC-IndustrialN_2_, AC-Mixed, AC- Urban, AC-Oily and AC-Paper)	Acetamiprid, thiamethoxam, and imidacloprid	Synthetic water and hospital wastewater effluent	Adsorption	[Ads]: 0.3 g·L^−1^, 250 rpm, 25 °C; Pesticide: 50 ppm	AC-Industrial: Acetamiprid: 104.2 mg·g^−1^; Thiamethoxam: 137 mg·g^−1^; Imidacloprid: 119.9 mg·g^−1^. Less than 60 min	[66]
(Bio)polymer-based materials						
External shells of marine gastropods	Bifenthrin	Synthetic water	Adsorption	Flow rate: 8 mL·min^−1^; Bed height: 4 cm; Particle size: 50–100 µm; Bifenthrin: 20 mg·L^−1^; 22 °C; pH: 6.0	Adsorption capacity: 40.53 mg	[72]
Chitosan and zeolite-A	Acephate, omthosate, and methyl parathion	Synthetic water	Adsorption	[Ads]: 0.7 g·L^−1^; 20 °C; pH: 8.0; Pesticide: 200 mg·L^−1^	Acephate: 650.7 mg·g^−1^; Omthosate: 506.5 mg·g^−1^; Methyl parathion: 560.8 mg·g^−1^. In 480 min	[73]
Magnetic chitosan and activated carbon (MCS/AC) with metal–organic frameworks (MOFs) (MCS/AC@UiO-66)	Co(II), azo dye, and the pesticide imidacloprid	Synthetic water and real water	Adsorption	30 mg of adsorbent; pH: 6.5; Co(II): 10 mg·L^−1^; Azo dye: 15 mg·L^−1^; Imidacloprid: 6 mg·L^−1^.	Co(II): 44.50 mg.g^−1^; Azo dye: 62.10 mg.g^−1^; Imidacloprid: 25.23 mg·g^−1^. In 15 min. Real samples: removal >90%	[74]
Chitosan with graphene quantum dots and iron oxide (CS/GQD-Fe_3_O_4_)	Chlorpyrifos	Synthetic water	Adsorption	[Ads]: 2 g·L^−1^; pH: 5.0; Chlorpyrifos: 30 ppm.	99.3%. In 50 min, or 10 min with ultrasonic	[75]
Three dead roots of invasive species and coffee and green tea grounds	Chlordecone, lindane, diuron, and ametryne	Synthetic water	Adsorption	Pollutants: 10 µmol·L^−1^. Chlordecone: [Ads]: 3 g·L^−1^; Lidane: [Ads]: 5 g·L^−1^; Diuron: [Ads]: 5 g·L^−1^; Ametryne: [Ads]: 5 g·L^−1^	Chlordecone: coffee grounds 96%; Lidane: coffee grounds 88%; Diuron: *F. japonica* TT 90%; Ametryne: coffee grounds 50%. In 2 h	[76]
Carboxymethyl cellulose, tryptophan, and titanium dioxide	2,4-dichlorophenol (2,4-DCP)	Synthetic water	Catalysis	0.5 g·L^−1^ catalyst; 2,4-DCP: 200 ppm; UV light; 25 °C	Degradation rate: 0.02061 min^− 1^	[77]
Other materials						
Zr(IV) loaded ligand exchange fibrous	Arsenic(V)	Synthetic water	Adsorption	pH: 2.01; As(V): 2.025 ppm	0.124 mmol·g^−1^	[88]
Mesoporous organosilica	Hg^2+^ and Cu^2+^	Synthetic water	Adsorption	[Ads]: 0.1 g·L^−1^; pH: 6; 25 °C; Metal ions: 100 ppm	Hg^2+^: 166 mg·g^−1^; Cu^2+^: 153 mg·g^−1^. In 30 min	[70]
Agricultural polyethylene film and wheat straw (APE, SCC, WSP, and WSS)	Malathion, diflubenzuron, difenoconazole, and carbendazim	Synthetic water	Adsorption	[Ads]: 10 g·L^−1^; 298 K	Malathion: WSP: 33.22 µg·g^−1^; Diflubenzuron: SCC: 500 µg·g^−1^; Difenoconazole: WSP: 188.68 µg·g^−1^; Carbendazim: WSS: 62.11 µg·g^−1^. In 120 min	[27]
Modified *Tenebrio molitor frass* biochar	Neonicotinoid pesticides (dinotefuran, DIN; nitenpyram, NIT; thiacloprid, THI)	Synthetic water	Adsorption	40 mg of adsorbent; pH: 7; 180 rpm; Pesticides: 100 mL	DIN: 165.28 mg·g^−1^; NIT: 223.14 mg·g^−1^; THI: 393.69 mg·g^−1^. In 200 min	[95]
Sugarcane bagasse (SCB) and beet pulp (BP)	Manganese	Synthetic water	Adsorption	1.5 g for SCB; 1.0 g for BP; pH: 6.0; Mn(II): 2 mg·L^−1^	SCB: 0.417 mg·g^−1^, in 150 min; BP: 0.864 mg·g^−1^. In 90 min	[96]
Activated and non-activated wood sawdust (SD and ASD)	Copper	Synthetic water	Adsorption	[Ads]: 5 g·L^−1^; ASD pH: 5; SD pH: 4; 20 °C; 200 rpm; Cu(II): 50 mg·L^−1^	ASD: 13.495 mg·g^−1^	[97]
Commercial coffee waste (UCR and TCR)	Copper and chrome	Synthetic water	Adsorption	[Ads]: 1 g·L^−1^; pH: 5; 140 rpm; 25 °C; Ion: 50 mg·L^−1^	Cu(II): TCR: 69 mg·g^−1^. Cr(VI): TCR: 44 mg·g^−1^. In 3 h	[98]
Adsorbent based on gum arabic and polyamidoxime	Chlorpyrifos	Synthetic water	Adsorption	0.005 g of adsorbent; pH: 6.0; 298 K; Chlorpyrifos: 300 ppm	769.23 mg·g^−1^. In 20 min	[79]
Pt@MWCNT/ZnTiO_3_	Thiamethoxam	Synthetic water	Catalysis	[Cat]: 1 g·L^−1^; 250 W visible light; Thiamethoxam: 20 ppm	92.62%. In 40 min	[80]
SnS_2_/CO_3_^2−^@Ni-Co LDH (SnS_2_/NCL)	Thiamethoxam	Synthetic water and real water	Catalysis	Catalyst: 0.16 g·L^−1^; 0.3 mM H_2_O_2_; Visible light; Thiamethoxam: 10 ppm	98.1%. In 70 min	[81]
Biotite modified with double hydroxide layer	Copper, lead, glyphosate (GLY), and 2-methyl-4-chlorophenoxyacetic acid (MCP)	Soil	Adsorption	20 mg of adsorbent; 25 °C; 200 rpm; pH: 7.0; Heavy metals: 320 mg·kg^−1^; Pesticides: 120 mg·kg^−1^.	GLY: 204.08 mg·g^−1^; MCP: 101.01 mg·g^−1^	[83]
*Eucalyptus sheathiana* bark	Zinc	Synthetic water	Adsorption	20 mg of adsorbent; pH: 5.1; 120 rpm; 30 °C; Zn^2+^: 200 ppm	Modified eucalyptus bark: 250 mg·g^−1^. In 2 h	[84]
Vineyard pruning wastes	Clothianidin (CTD), Acetamiprid (ACE), 2,4-D, Metalaxyl (MET), and Atrazine (ATZ)	Synthetic water	Adsorption	100 mg of adsorbent; pH 7.0; Pesticides: 20 µmol·L^−1^	CTD: 85%; ACE: 94%; MET: 86%; ATZ: 95%; 2,4-D: 4%	[67]
Banana peels	Ethoprophos (ETH), Terbufos (TER), and Diazinon (DIA)	Synthetic water	Adsorption	70 mg of adsorbent; pH: 4.0; 25 °C; Insecticides: 1.5 ppm	ETH: 3.19 mg·g^−1^; TER: 3.07 mg·g^−1^; DIA: 3.19 mg·g^−1^. In 3 h	[68]
*Prunus serrulata*	Atrazine	Synthetic water and real water	Adsorption	[Ads]: 0.8 g·L^−1^; pH: 3.0; 328 K; Atrazine: 50 ppm	63.35 mg·g^−1^. In 240 min	[69]
Tungsten boride iron phosphate (FePO_4_/WB-PMS)	Imidacloprid (IMI), Nitenpyram (NIT), Thiacloprid (THI), Dinotefuran (DIN), And Clothianidin (CLO)	Synthetic water and real water	Catalysis	[Cat]: 250 ppm; pH: 6.78; 25 °C; NEO: 10 ppm	IMI: ≈100%; THI: ≈100%; NIT: 96.1%; DTN: 89%; CLO: 82%. In 50 min	[85]
Fe_3_O_4_@G-TEOS–MTMOS	Phosphamidon, dimethoate, chlorpyrifos, and diazinon	Environmental water	Adsorption	80 mg of adsorbent; pH: 7.0	Phosphamidon: 54.35 mg·g^−1^; Dimethoate: 58.14 mg·g^−1^; Chlorpyrifos: 37.18 mg·g^−1^; Diazinon: 76.34 mg·g^−1^	[82]

**Table 3:** Comparison of the material types discussed in this work.

Material	Examples	Advantages	Disadvantages	Efficiency	Ref.
Agro-industrial waste	Rice husk biochar, sugarcane bagasse ash, and activated carbon from coffee grounds	Low cost, sustainable, good adsorption capacities, and eco-friendly	Variability in composition and potential for inconsistent performance	Moderate/High	[30]
Carbonaceous	Activated carbon, biochar, carbon nanotubes, and graphene	High surface area, good adsorption capacity, and versatile	Production cost	High	[7]
Clay-based	Kaolinite, montmorillonite, bentonite, and illite	High adsorption capacity, abundant, and efficient for various contaminants	Require modification for optimal performance	High	[49]
Biopolymer-based	Chitosan and sodium alginate	Biodegradable, renewable, and high adsorption capacity	Complex synthesis and limited stability in certain conditions	Moderate/High	[100]
Other	Mesoporous silica, hydrogels, and hybrid materials	High efficiency, tunable properties, and effective for various pollutants	Complex synthesis and high cost	Moderate/High	[80]

## Data Availability

Not applicable.
